# Analysis of the Temporo-Spatial and Electromyographic Characteristics of Gait in a Hemiplegic Patient: A Single-Subject Case Report

**DOI:** 10.3390/reports9010006

**Published:** 2025-12-24

**Authors:** Nohra Fernanda Nuñez Molano, Daniela Scarpetta Castrillon, Florencio Arias Coronel

**Affiliations:** 1 Faculty of Health, Universiadad Santiago de Cali, Cali 760035, Colombia; nohra.nunez00@usc.edu.co (N.F.N.M.); daniela.scarpetta00@usc.edu.co (D.S.C.); 2 Grupo de Investigacion GISI, Universidad Santiago de Cali, Cali 760035, Colombia

**Keywords:** surface electromyography, inertial sensors, hemiplegia, gait, stroke, motion analysis, neurorehabilitation

## Abstract

**Background and Clinical Significance:** Hemiplegia following a cerebrovascular accident (CVA) disrupts gait symmetry and efficiency, compromising functional independence. The integration of surface electromyography (sEMG) and inertial measurement units (IMU) enables quantitative assessment of muscle activation and segmental dynamics, providing objective data for therapeutic planning. **Case presentation**: A 57-year-old male with chronic right hemiplegia, eight years post-ischemic stroke of the left middle cerebral artery. The patient ambulated independently without assistive devices, exhibiting right lower-limb circumduction. Clinical assessment revealed the following scores: Barthel Index 85/100, Tinetti Performance-Oriented Mobility Assessment (POMA) 16/28, Timed Up and Go (TUG) test 13 s, and Modified Ashworth Scale (MAS) scores of 1 (upper limb) and 1+ (lower limb). Methods: Multichannel sEMG (Miotool 800^®^, 8 channels) was recorded form the lumbar erectors, gluteus medius and maximus, vastus medialis, vastus intermedius, vastus lateralis, biceps femoris, tibialis anterior, medial gastrocnemius, and lateral gastrocnemius. Ag/AgCI electrodes were positioned according to SENIAM recommendations: sampling rate: 1000 Hz; band-pass filter: 20–500 Hz; notch filter: 60 Hz; normalization to %MVC. Simultaneously, IMU signals (Xsens DOT^®^, 60 Hz) were collected from both ankles during slow, medium and fast walking (20 s each) and compared with a healthy control subject. Results: The patient exhibited reduced sEMG amplitude and increased peak irregularity on the affected side, particularly in the gluteus medius, tibialis anterior, and gastrocnemius, along with agonist desynchronication. IMU data revealed decreased range of motion and angular pattern irregularity, with inconsistent acceleration peaks in the right ankle compared to the control, confirming neuromuscular and kinematic asymmetry. **Conclusions**: The combined sEMG-IMU analysis identified deficits in selective motor control and propulsion on the affected hemibody, providing essential information to guide physiotherapeutic interventions targeting pelvic stability, dorsiflexion, and propulsive phase training, enabling objective follow-up beyond specialized laboratory settings.

## 1. Introduction and Clinical Significance

Stroke is one of the leading causes of motor disability worldwide and frequently results in hemiplegia, which alters gait patterns and postural control. The resulting motor and sensory deficits impair gait symmetry, coordination, and efficiency, increasing the risk of falls and reducing functional independence [1,2,3]. In this context, instrumental gait analysis has become an essential tool for the objective assessment of motor patterns in patients with neurological impairments [4,5].

Post-stroke spasticity is a common complication, affecting up to one-third of survivors, and is characterized by a velocity-dependent increase in muscle tone [6]. Spasticity interferes with selective motor control, joint mobility, and gait symmetry, and it represents a major contributor to abnormal gait patterns and functional limitations during ambulation. Globally, it is estimated that approximately 11.9 million new strokes occur each year, and around 93.8 million individuals live with stroke-related sequelae. In Colombia, cerebrovascular diseases rank among the main causes of death and disability; for instance, the mortality rate from stroke reached approximately 32.45 deaths per 100,000 inhabitants in 2023, corresponding to about 16,946 deaths [7]. This high population burden highlights the need for objective functional assessment tools, such as surface electromyography and inertial sensors, to optimize rehabilitation strategies in patients with motor impairments.

Surface electromyography (sEMG) enables the non-invasive recording of muscle electrical activity, providing detailed information on the timing and intensity of activation throughout the gait cycle [8]. Complementarily, inertial measurement units (IMUs) offer an accessible method to quantify acceleration and angular motion parameters in real time, facilitating quantitative analysis outside highly specialized laboratories [9,10].

The integration of these two technologies provides a comprehensive view of neuromuscular and kinematic behavior, allowing the detection of asymmetries, co-activations, and motor control disturbances in post-stroke patients. Their application in case reports contributes to evidence-based clinical practice and supports the design of individualized therapeutic strategies.

**Clinical relevance:** This case highlights the clinical usefulness of combined sEMG–IMU recording to identify abnormalities in muscle activation and gait dynamics in a patient with chronic hemiplegia, providing objective data to guide individualized physiotherapeutic planning.

## 2. Case Presentation

The case involves a 57-year-old male resident of Cali, Colombia, diagnosed with right hemiparesis secondary to an ischemic stroke affecting the left middle cerebral artery territory, which occurred approximately eight years ago. The patient holds a certified physical motor disability status and has a history of receiving outpatient physiotherapy during the subacute phase.

At the time of the current evaluation, the patient ambulated independently without assistive devices but exhibited an asymmetric gait pattern, characterized by circumduction of the right lower limb, a wide base of support, and trunk compensation toward the non-affected side. He reported no recent falls and demonstrated adequate comprehension and cooperation during both clinical and instrumental assessments.

Neuromuscular and functional evaluations were conducted at the Gait Analysis Laboratory of Universidad Santiago de Cali, under controlled environmental conditions (temperature, lighting, and safety). Standardized scales were administered to assess functional independence, balance, muscle tone, and gait performance.

(Table 1) The patient showed mild dependence (Barthel Index: 85/100) and a high risk of falls according to the Tinetti Scale (16/28). Mild spasticity was observed in the right hemibody, with a tone score of 1+ on the Modified Ashworth Scale. In the Timed Up and Go (TUG) test (13 s), the patient demonstrated independent gait with slight slowness but no loss of balance. Muscle strength was reduced in the affected hemibody (grade 2+), while the contralateral side exhibited normal strength (grade 5), confirming the presence of motor asymmetry.

The Timed Up and Go (TUG) test evaluates functional mobility by measuring the time required to stand up from a chair, walk 3 m, turn, and return to the seated position; values below 14 s typically indicate functional independence. The Tinetti Performance-Oriented Mobility Assessment evaluates balance and gait on a 28-point scale, where scores below 19 suggest a high risk of falls. The Modified Ashworth Scale (MAS) measures spasticity based on resistance to passive muscle stretch, with scores ranging from 0 (no increase in tone) to 4 (rigid limb), while the grade 1+ indicates a slight increase in muscle tone through less than half of the range of motion.

**Table 1:** **Functional clinical assessment of the patient with right hemiplegia**.

Test/Scale	Result	Clinical Interpretation
Barthel Index	85/100	Mild dependence
Tinetti Scale	16/28	High risk of falls
Modified Ashworth Scale	1 (Upper limb), 1 + (Lower limb)	Mild increase in muscle tone on the right hemibody
Timed Up and Go (TUG)	13 s	Independent gait with slight slowness, no loss of balance

### 2.1. Electromyographic Signal Acquisition (sEMG)

Muscle electrical activity was recorded using surface electromyography (sEMG) with the Miotool 800^®^ system (Miotec, Brazil), configured with eight channels. Due to this 8-channel limitation, bilateral muscle recordings were obtained in sequential acquisition sets rather than simultaneously for all muscles. Prior to recording, the skin was prepared using sterile gauze and 70% isopropyl alcohol to optimize electrical conductivity and reduce skin–electrode impedance.

Ag/AgCl self-adhesive electrodes were positioned according to the SENIAM (Surface Electromyography for the Non-Invasive Assessment of Muscles) recommendations, placed longitudinally along the muscle fibers with an inter-electrode distance of 2 cm. Cables were secured with hypoallergenic tape to minimize motion artifacts.

The muscles selected for recording were:

- Lumbar erectors
- Gluteus medius
- Gluteus maximus
- Vastus lateralis
- Vastus intermedius
- Vastus medialis
- Biceps femoris
- Tibialis anterior
- Medial gastrocnemius
- Lateral gastrocnemius

sEMG signals were acquired at a sampling frequency of 1000 Hz, with band-pass filters between 20 and 500 Hz and a 60 Hz notch filter to eliminate power line interference. Signal quality was visually inspected before initiating formal data acquisition.

The gait protocol included three controlled-speed conditions:

- Slow gait
- Medium gait
- Fast gait

(Table 2) Each condition was recorded for 20 s on a treadmill under professional supervision, ensuring patient safety and stability of the gait pattern during acquisition. The same protocol was applied to a healthy control subject of similar age and body composition, in order to enable bilateral and inter-subject comparison.

Comparison of bilateral electromyographic activity was performed for the gluteus medius, vastus lateralis, tibialis anterior, and medial gastrocnemius muscles between the patient with right hemiparesis and the healthy control.

**Table 2:** Bilateral electromyographic comparison between the hemiparetic patient and the control subject at three gait speeds: (I) slow (0–20 s), (II) medium (20–40 s), and (III) fast (40–60 s). Representative signals of the recorded muscles (M): (a) gluteus medius, (b) vastus lateralis, (c) tibialis anterior, and (d) medial gastrocnemius. In the hemiparetic patient, reduced amplitudes and irregular peak patterns are observed, in contrast to the rhythmic and sustained activation seen in the control subject.

M	V	Patient with Hemiparesis	M	V	Healthy Subject (Control)
(a)		Gluteus medius	(a)		Gluteus medius
	(I)	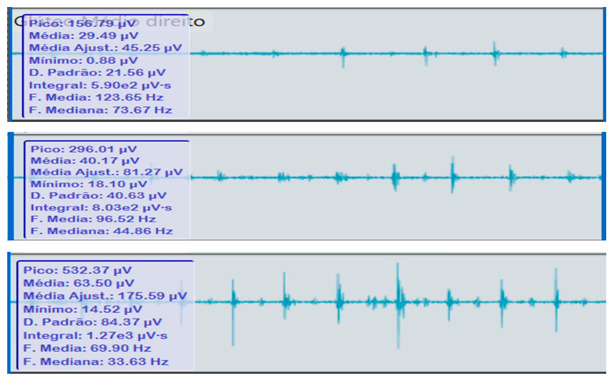		(I)	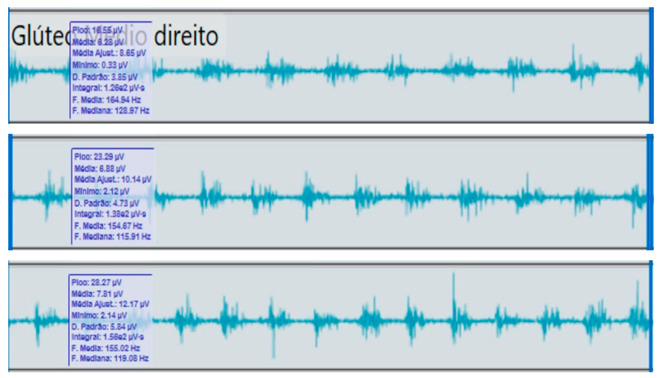
	(II)			(II)	
	(III)			(III)	
(b)		Vastus lateralis	(b)		Vastus lateralis,
	(I)	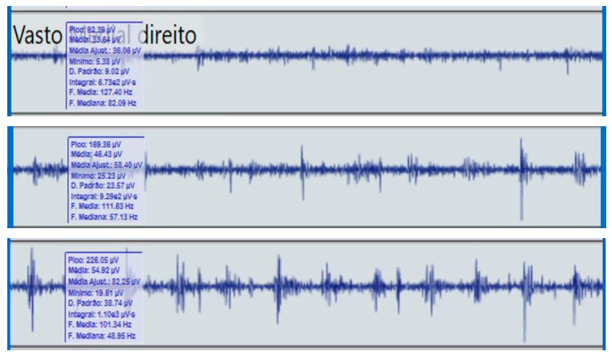		(I)	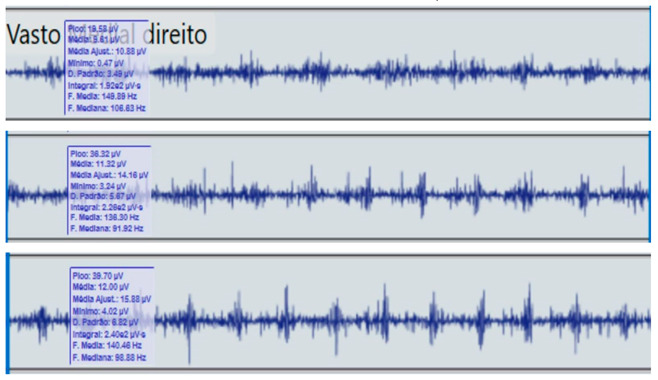
	(II)			(II)	
	(III)			(III)	
(c)		Tibialis anterior	(c)		Tibialis anterior
	(I)	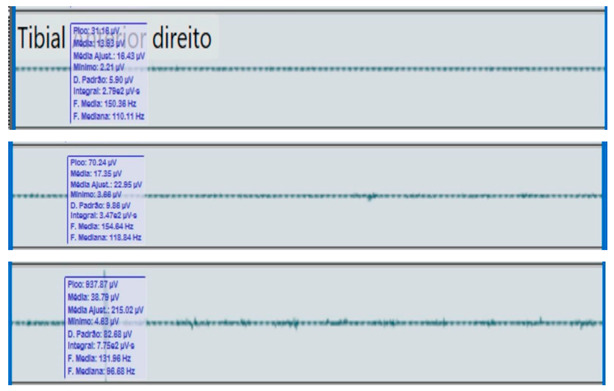		(I)	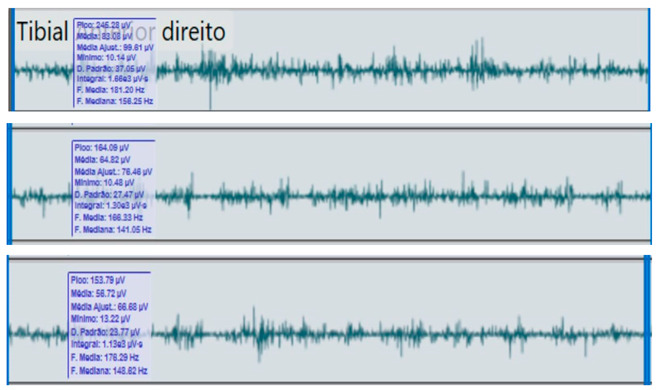
	(II)			(II)	
	(III)			(III)	
(d)		Medial gastrocnemius	(d)		Medial gastrocnemius
	(I)	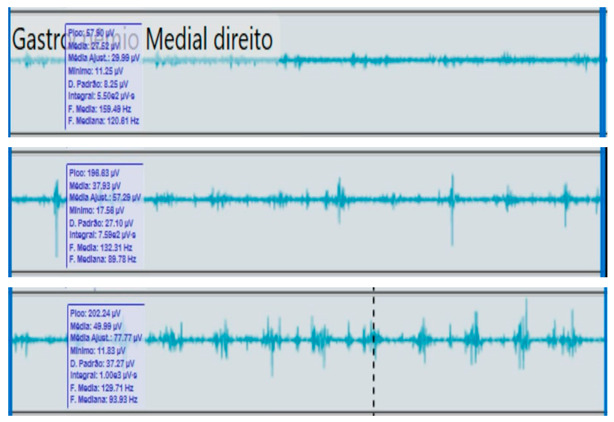		(I)	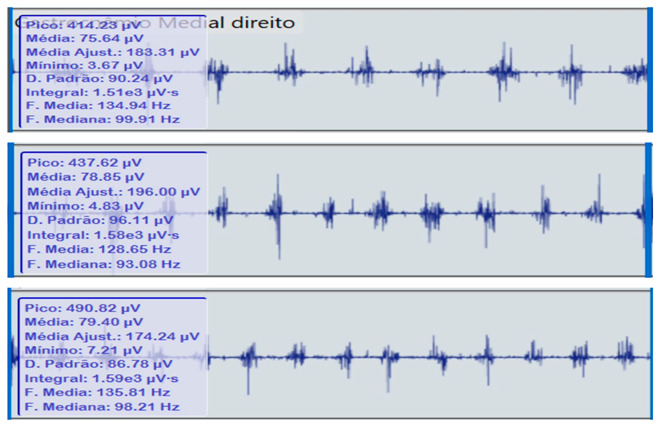
	(II)			(II)	
	(III)			(III)	

### 2.2. Calibration and Signal Normalization

Before data acquisition, electrode impedance (<5 kΩ) was verified to ensure recording quality. Signal normalization was performed using the maximum voluntary contraction (MVC) of each muscle, obtained from brief isometric contractions in a stable standing position.

The values recorded during gait were expressed as a percentage of the peak amplitude (%MVC), allowing comparison of muscle activation between gait speeds and body sides. The signals were visually inspected to identify and remove motion artifacts, electrical interference, or inadequate electrode contact, ensuring the quality and integrity of the recordings prior to final analysis.

### 2.3. Inertial Sensor (IMU) Recording

Simultaneously with the electromyographic recordings, Xsens DOT^®^ inertial sensors were placed on the anterior region of both ankles and configured in high-fidelity mode (60 Hz). The devices captured linear acceleration (m/s^2^) and angular velocity (°/s) during the three gait conditions: slow, medium, and fast (20 s each).

**Treadmill speeds were standardized as follows: slow = 0.6 m/s, medium = 0.9 m/s, and fast = 1.2 m/s.**

IMU signals were temporally synchronized with the sEMG data, allowing correlation of acceleration and deceleration peaks with muscle activation during each phase of the gait cycle.

Although EMG signals were sampled at 1000 Hz and IMU signals at 60 Hz, temporal alignment of both datasets was performed during post-processing. This procedure ensured accurate synchronization of gait events and allowed consistent comparison between muscular activation and segmental kinematics.

The patient tolerated all test conditions without adverse events or fatigue. Subsequently, the data were exported for comparative analysis with a healthy control subject of similar anthropometric characteristics.

### 2.4. Comparative Electromyographic Activity

Table 3 presents the mean and peak (µV) values of the electromyographic activity recorded from the main muscles of the right and left lower limbs in the patient with hemiplegia, compared with a healthy control subject of similar characteristics.

The recordings were obtained during slow, medium, and fast gait conditions, with all values normalized to the percentage of the maximum voluntary contraction (%MVC).

**Table 3:** Comparative electromyographic activity of the main lower limb muscles during gait.

Muscle	Speed	Right LL/Hemiparetic Side (Mean/Peak)	Left LL/Non-Affected Side (Mean/Peak)	Right LL/Control Subject (Mean/Peak)	Left LL/Control Subject (Mean/Peak)
Lumbar erectors	Slow	32.4/89.5	44.0/110.2	46.0/115.6	45.2/113.9
	Medium	38.7/98.1	48.3/118.4	50.1/121.3	49.6/120.7
	Fast	42.9/105.3	52.1/124.6	55.4/129.8	54.9/129.0
Gluteus medius	Slow	41.2/108.6	76.3/151.2	82.4/160.5	85.1/165.7
	Medium	54.8/125.9	88.5/168.4	91.2/174.6	92.5/180.2
	Fast	65.1/140.7	92.1/175.9	97.8/182.3	99.6/188.0
Gluteus maximus	Slow	38.7/95.4	70.2/141.6	77.9/149.0	80.3/152.8
	Medium	49.3/116.2	83.5/156.4	89.1/165.2	91.4/170.0
	Fast	57.8/130.5	89.4/162.3	95.8/173.5	98.7/178.4
Vastus lateralis	Slow	51.8/119.3	82.5/155.4	88.6/161.2	90.3/165.7
	Medium	63.2/132.7	89.8/164.5	94.1/170.1	95.4/174.9
	Fast	72.1/144.0	94.7/172.3	98.2/178.5	99.8/182.9
Vastus intermedius	Slow	49.5/112.0	79.1/150.3	84.8/157.9	86.2/160.5
	Medium	59.8/128.6	87.2/161.8	91.9/168.7	93.6/172.2
	Fast	69.3/138.9	91.5/169.0	96.0/175.4	98.1/179.8
Vastus medialis	Slow	46.2/105.4	76.8/147.9	82.5/155.2	84.0/157.6
	Medium	57.4/121.5	84.6/159.3	89.1/166.8	91.5/170.4
	Fast	67.1/134.2	89.3/165.6	93.7/172.0	96.0/176.5
Biceps femoris	Slow	35.6/90.1	61.2/127.4	66.8/136.2	68.1/139.5
	Medium	47.5/108.3	73.9/142.0	78.2/150.1	80.4/153.2
	Fast	56.2/119.9	82.7/151.3	87.6/158.7	89.3/162.0
Tibialis anterior	Slow	43.8/101.5	70.4/135.2	75.9/144.7	77.3/147.8
	Medium	54.2/115.8	81.3/142.1	85.7/150.6	87.0/153.9
	Fast	62.9/128.3	88.6/153.8	92.5/160.2	94.3/164.5
Medial gastrocnemius	Slow	39.4/98.2	58.7/132.4	66.9/144.0	67.8/146.2
	Medium	48.1/112.6	72.3/145.7	79.1/152.4	80.5/154.8
	Fast	57.6/126.8	81.5/158.1	88.6/162.0	90.2/166.3
Lateral gastrocnemius	Slow	37.8/95.1	56.5/129.3	64.1/140.8	65.5/143.0
	Medium	46.9/110.9	69.7/143.8	77.2/150.3	79.0/153.1
	Fast	55.4/123.6	79.8/155.9	85.4/159.8	87.6/163.4

During gait, the patient showed a significant reduction in electromyographic amplitude on the right hemibody, particularly in the gluteus medius, tibialis anterior, and medial gastrocnemius, compared with the unaffected side and the control subject. The contralateral and control patterns remained symmetrical and speed-modulated, indicating more efficient neuromuscular recruitment. These findings reflect activation deficits and compensatory coactivation that compromise pelvic stability, propulsion, and postural control.

### 2.5. Clinical Qualitative Analysis of EMG

The qualitative analysis of the electromyographic signals allowed clear identification of differences in amplitude, frequency, and synchronization of muscle activation between the patient with right hemiplegia and the control subject.

The right gluteus medius showed low amplitude (<100 µV) and irregular frequency, with delayed activation during the stance phase, associated with pelvic instability and contralateral trunk compensation.

The vastus lateralis exhibited intermittent activity and asynchrony with the hamstrings, reflecting a deficit in intermuscular coordination and decreased propulsive efficiency.

The tibialis anterior demonstrated reduced amplitude (<60 µV) and periods of inactivity during the toe-off phase, which correlated with foot drag and impaired distal control.

The medial gastrocnemius showed weak contractions (70–80 µV) without clearly defined peaks during the push-off phase, indicating a low contribution to the final propulsion.

The hamstrings displayed desynchronization with the rectus femoris, evidencing impaired agonist–antagonist coordination.

In contrast, the control subject exhibited regular, rhythmic, and well-coordinated electromyographic patterns (140–180 µV), with sustained and symmetrical activations in both lower limbs, consistent with stable physiological gait.

**Comparative clinical conclusion:**

The hemiplegic patient presented weak, asynchronous, and disorganized activations in the right hemibody, mainly in the gluteus medius, tibialis anterior, and gastrocnemius, resulting in pelvic instability, foot drag, and loss of propulsion. In contrast, the control subject showed a stable and efficient electromyographic pattern consistent with preserved neuromuscular control.

### 2.6. Analysis of Inertial Data (IMU)

Complementarily, the analysis of signals obtained from the Xsens DOT^®^ inertial sensors enabled evaluation of the segmental dynamics of both ankles during slow, medium, and fast gait. The results were expressed as angular variations in the pitch axis, temporally synchronized with the muscular activations recorded by sEMG.

To improve clarity and facilitate interpretation, the IMU signals of the hemiparetic patient and the healthy control subject are presented separately in the updated figures, as suggested by the reviewer.

**Figure 1:**
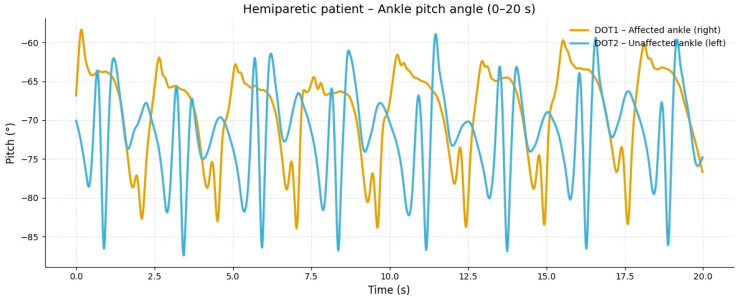
**Slow-speed gait (0–20 s).** At slow speed, the affected limb (right ankle) showed reduced angular excursion and irregular oscillation patterns compared to the healthy side. The limited pitch amplitude indicates lower propulsion and insufficient activation of the dorsiflexor and plantar flexor muscles during stance and swing phases.

In the hemiparetic patient, during slow-speed gait (0–20 s), the affected limb showed reduced oscillation amplitude and irregular angular cycles compared to the non-affected side, reflecting impaired distal motor control (Figure 1).

**Figure 2:**
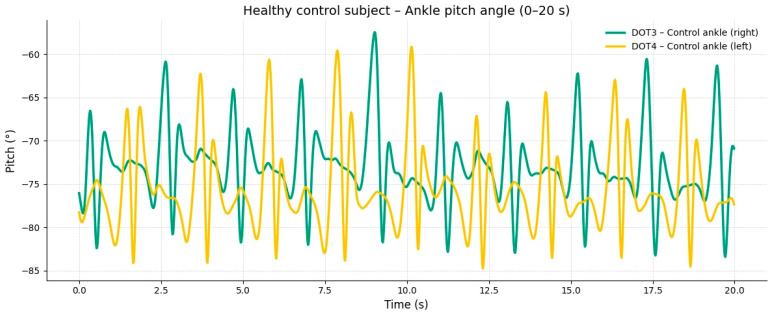
**Moderate-speed gait (20–40 s).** At intermediate speed, an increase in the amplitude and frequency of oscillation was observed in both ankles; however, the affected limb maintained an asymmetric pattern with inconsistent acceleration peaks. These findings reflect excessive coactivation between quadriceps and hamstring muscles, resulting in stiffness and reduced smoothness of the gait cycle.

The corresponding signals recorded from the healthy control subject at the same walking speed showed regular, symmetric, and stable pitch oscillations in both ankles (Figure 2).

**Figure 3:**
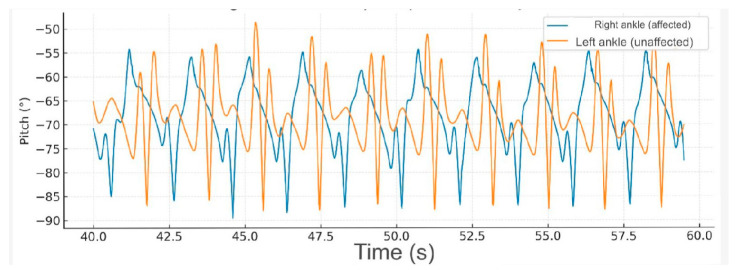
**Fast-speed gait (40–60 s).** At higher speed, the amplitude of ankle movement increased bilaterally, but the hemiparetic limb continued to demonstrate delayed peaks and irregular rhythmicity. This pattern suggests impaired motor control and compensatory recruitment of proximal muscles to sustain gait velocity.

At medium-speed gait (20–40 s), the hemiparetic patient exhibited an increase in angular amplitude and frequency in both ankles; however, the affected limb maintained an asymmetric pattern with delayed and inconsistent peaks, indicating persistent alterations in motor coordination (Figure 3).

**Figure 4:**
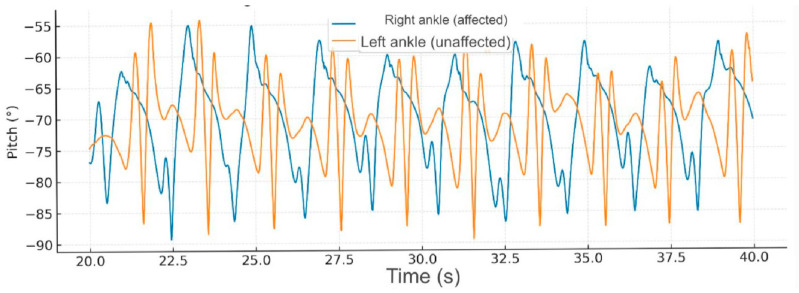
**Comparative gait kinematics between the hemiparetic patient and the healthy control (0–60 s).** The control subject exhibited consistent, symmetric oscillations across all gait cycles, while the hemiparetic patient presented marked asymmetry, reduced pitch amplitude, and irregular temporal intervals between steps.

During fast-speed gait (40–60 s), further increases in ankle pitch amplitude were observed; nevertheless, the hemiparetic limb continued to present instability and abrupt angular variations, consistent with reduced distal control and altered activation of the gluteus medius and gastrocnemius muscles (Figure 4).

## 3. Discussion

The present case demonstrates the usefulness of the combined analysis of surface electromyography (sEMG) and inertial measurement units (IMU) in identifying neuromuscular and kinematic alterations in a patient with chronic post-stroke hemiparesis. The findings revealed a marked reduction in electromyographic amplitude in the right hemibody, particularly in the gluteus medius, tibialis anterior, and medial gastrocnemius, accompanied by an irregular angular pattern of the affected ankle in the pitch axis. This correlation reinforces the relationship between muscle weakness, intersegmental discoordination, and the loss of postural control during gait, clinically observed as limb circumduction and reduced propulsion [11].

These results are consistent with previous reports highlighting muscle activation asymmetry and reduced selective motor control in post-stroke patients, which result in a less efficient and more energetically demanding gait pattern [12,13]. Allen et al. and Fujita et al. demonstrated that post-stroke electromyographic asynchrony during gait leads to contralateral compensations and negatively affects movement economy [14,15].

Several studies have described the gluteus medius and tibialis anterior as key muscles for pelvic stability and foot control during the stance phase. Their reduced activation leads to trunk compensations and widening of the base of support. Likewise, diminished medial gastrocnemius activity compromises the final propulsive phase, reducing the ability to generate impulse and walking speed [16,17].

These findings are consistent with previous research describing characteristic neuromuscular deficits in post-stroke gait. Pradon et al. reported reduced activation of the tibialis anterior and delayed onset of the gastrocnemius in hemiparetic patients, contributing to foot drop and decreased push-off, similar to the alterations observed in our case. Likewise, Kim and Eng demonstrated that weakened gluteus medius activation disrupts pelvic stability, increasing compensatory trunk lean, which aligns with the asymmetrical kinematic pattern identified by the IMU in this study. Furthermore, recent studies highlight that combining EMG and IMU data enhances the detection of subtle gait abnormalities and improves clinical interpretation by capturing both muscular and segmental deficits simultaneously.

The use of IMU sensors allowed an objective quantification of the altered angular pattern, complementing the electromyographic information and facilitating a better understanding of the underlying biomechanical mechanisms. Recent studies confirm that IMUs are valid and reliable tools for kinematic gait analysis, even in patients with neuromotor impairments [18,19].

The combination of both methods offers an accessible and accurate alternative for clinical gait monitoring in non-specialized environments, contributing to a more comprehensive and personalized functional assessment [20,21]. From a therapeutic perspective, these findings suggest the need for physiotherapeutic interventions focused on neuromuscular re-education, selective activation of stabilizing muscles (gluteus medius and tibialis anterior), and distal propulsion training through visual and sensory feedback strategies [22].

Although no physiotherapeutic intervention was performed as part of this study, the objective characterization of gait using combined sEMG–IMU analysis provides clinically meaningful information that may guide rehabilitation planning. By identifying specific impairments—such as reduced selective motor control, impaired pelvic stability, decreased ankle dorsiflexion, and limited propulsive force—the results of this assessment can support clinicians in selecting targeted therapeutic exercises and neuromuscular re-education strategies. Therefore, this case report contributes to the growing body of evidence suggesting that the integration of sEMG and IMU technologies can enhance clinical decision-making and improve individualized rehabilitation approaches in post-stroke gait dysfunction.

Overall, this case reaffirms the relevance of the combined sEMG–IMU approach as an objective tool for the evaluation and follow-up of patients with hemiparesis, providing quantitative information that complements clinical observation and guides individualized therapeutic decision-making [23].

This case report has several limitations, including its single-subject design, which limits generalizability, and the absence of kinetic data or three-dimensional gait analysis. However, it also presents important strengths: the combined use of sEMG and IMU provides detailed neuromuscular and kinematic information that is rarely integrated in clinical case reports, offering a practical and accessible tool for clinicians to objectively evaluate gait impairments in post-stroke individuals.

## 4. Conclusions

The combined analysis of surface electromyography and inertial sensors objectively identified neuromuscular and kinematic alterations in a patient with chronic post-stroke hemiparesis.

The reduced electromyographic amplitude, together with the angular asymmetry recorded in the pitch axis, revealed deficits in selective motor control, intersegmental discoordination, and decreased gait efficiency.

These findings confirm the clinical value of simultaneous sEMG–IMU recording as a complementary tool for monitoring the functional progression of neurological patients, providing quantitative data that strengthen clinical interpretation and physiotherapeutic planning.

Its application supports objective monitoring of therapeutic progress and the individualization of intervention strategies focused on pelvic stability reeducation, distal propulsion, and postural control.

Overall, this case highlights the importance of integrating accessible and portable technologies into clinical settings to optimize functional diagnosis and rehabilitation decision-making.

## Data Availability

The original contributions presented in this study are included in the article. Further inquiries can be directed to the corresponding author.
